# Induced membrane technique combined with a retrograde intramedullary nail for the treatment of infected bone defects of the ankle

**DOI:** 10.1038/s41598-023-34014-0

**Published:** 2023-04-24

**Authors:** Jingshu Fu, Xiaohua Wang, Shulin Wang, Zenggang Chen, Jie Shen, Zhengyun Li, Zhao Xie

**Affiliations:** 1 Department of Orthopedics, Banan Hospital of Chongqing Medical University, Chongqing, 401320 People’s Republic of China; 2 National & Regional United Engineering Laboratory of Tissue Engineering, Department of Orthopaedics, First Affiliated Hospital, Third Military Medical University (Army Medical University), Chongqing, 400038 People’s Republic of China

**Keywords:** Trauma, Bone

## Abstract

In this study, we treated infected ankle bone defects with the induced membrane two-stage technique. The ankle was fused with a retrograde intramedullary nail in the second stage, and the aim of this study was to observe the clinical effect. We retrospectively enrolled patients with infected bone defects of the ankle admitted to our hospital between July 2016 and July 2018. In the first stage, the ankle was temporarily stabilized with a locking plate, and antibiotic bone cement was used to fill the defects after debridement. In the second stage, the plate and cement were removed, the ankle was stabilized with a retrograde nail, and tibiotalar-calcaneal fusion was performed. Then, autologous bone was used to rebuild the defects. The infection control rate, fusion success rate and complications were observed. Fifteen patients were enrolled in the study with an average follow-up of 30 months. Among them, there were 11 males and 4 females. The average bone defect length after debridement was 5.3 cm (2.1–8.7 cm). Finally, 13 patients (86.6%) achieved bone union without recurrence of infection, and 2 patients experienced recurrence after bone grafting. The average ankle-hindfoot function score (AOFAS) increased from 29.75 ± 4.37 to 81.06 ± 4.72 at the last follow-up. The induced membrane technique combined with a retrograde intramedullary nail for the treatment of infected bone defects of the ankle after thorough debridement is an effective treatment method.

## Introduction

High-energy trauma, such as traffic injury or falling from a height, often leads to skin and soft tissue necrosis, deformity, infection and other complications due to sparse soft tissue coverage of the ankle, and infection is the most difficult problem. Eradicating the infection, repairing bone defects, and maximizing limb function are challenges for orthopaedic surgeons^1^. Currently, commonly used methods, including the Ilizarov technique^2,3^, Masquelet technique^4,5^ and vascularized fibula transplantation^6,7^, have their own advantages and limitations.

Bone transport (Ilizarov technique) or the induced membrane technique (Masquelet technique) have been commonly used treatments for bone defects in recent years. Bone transport is a classic treatment that relies on the "stretch-stress principle" to stimulate tissue regeneration, it has been widely reported in the treatment of bone defects. The induced membrane technique is carried out in two stages, in the first stage, Polymethyl methacrylate(PMMA) spacer is inserted in the defects for the formation of induced membrane, in the second stage, grafts are implanted in the membrane to repair the bone defect. In recent years, the indications have expanded from initial traumatic bone defects to open fractures, nonunion, extremity bone infection, congenital prosthetic joints and tumours^4,8,9^.

Treatment of infected bone defects of the ankle is a great challenge for orthopaedic surgeons, as eradication of infection is the most difficult challenge, and most ankle infections require ankle joint fusion after infection control. At present, the most common fixation method for infected bone defects of the ankle is external fixation^10–14^. There are very few reports about the combination of the induced membrane technique with retrograde intramedullary nails for the treatment of ankle infections^10–15^. Therefore, this study retrospectively analysed the clinical effect of the induced membrane technique combined with a retrograde intramedullary nail for the treatment of infected bone defects of the ankle after control of the infection.

## Methods

### General information

After approval by The Ethics Committee of the First Affiliated Hospital, Army Medical University, PLA (no: KY201878) and all the patients signed informed consent for the use of their information and images, all methods were performed in accordance with the relevant guidelines and regulations. A retrospective analysis of 15 patients with ankle infection admitted to our hospital between July 2016 and July 2018, included 11 males and 4 females with an average age of 47 years (29–68 years).

Inclusion criteria: patients with infected bone defects of the ankle; 18–70 years of age; treatment with the induced membrane two-stage technique; ability of the incision to be sutured directly or covered with a flap after the first stage; and fixation with retrograde intramedullary nail for ankle fusion in the second stage. Exclusion criteria included patients who did not receive surgical treatment; incomplete follow-up data; and presence of malignant tumours.

### Surgical technique

All patients were treated with two stages treatment: debridement until infection control in the first stage , retrograde intramedullary nail fixation combined with bone graft in the second stage.

In the first stage, the location of the infected lesion and the scope of resection were determined before surgery, which based on the patient's clinical signs and examinations such as X-ray, MRI and SPECT findings. The patient was placed in the supine position during operation, followed by an initial incision to the deep tissue and removal of the sinus. The lesion was completely removed and sent to isolate bacteria, fungi, and *Mycobacterium tuberculosis*. Next, hydrogen peroxide, diluted iodine, and a large amount of saline were pulsed to wash the wound. The field was resterilized; gloves, surgical gowns and surgical instruments were changed. The ankle and hind foot were adjusted along the force line at 5° valgus, 5° external rotation, and dorsiflexion-plantar flexion 0°, followed by stabilization with a locking plate. Antibiotic bone cement (Heraeus, Germany) was mixed with 5 g of vancomycin (Eli Lilly, USA) to fill the cavity and wrap the plate. When the cement was heated, it was cooled with cold saline, and a drainage tube was placed in the incision. The incision was sutured directly or covered with a flap. Empirical use of intravenously administered broad-spectrum antibiotics was applied, the antibiotics were adjusted after the bacteria were isolated based on sensitivity, and ambulation was allowed on the affected limb without full weight bearing.

**Figure 1:**
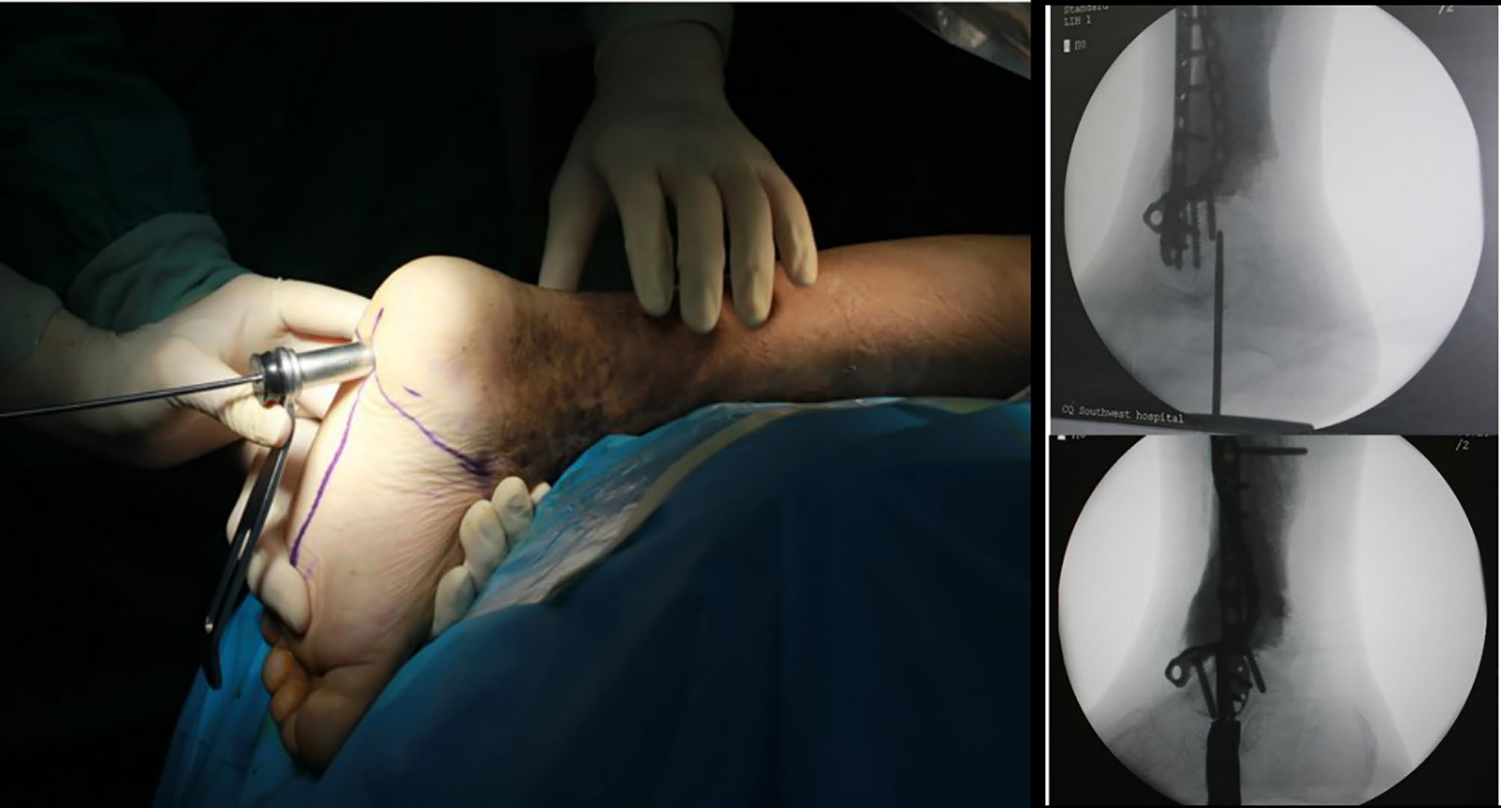
Key point to insert the nail. First, the entry point was identified and marked on the surface under fluoroscopy, and the original internal fixation and cement were removed after opening and reaming, which effectively reduced the fluoroscopy and operation time.

In the second stage, 6–8 weeks after the first stage, bone grafts were used to repair the defects. Before that, the patients had incision healing, along with no signs of bone infection, no fever, and no pain. Laboratory examinations of the white blood cell count, erythrocyte sedimentation rate and C-reactive protein were normal. The bone defects were approximately regarded as cylinders, detect the diameter and length of the defects with CT scan to calculate the amount of bone grafting required. The patient was instructed to bathe their feet with soapy water before the operation. The prone position or lateral position was adopted; after strict disinfection of the drape, an autograft was taken from one or both iliac crests, a sterilized tourniquet was placed on the base of the thigh, with a tourniquet pressure of 300 mmHg. According to the Stephenson body surface positioning method, a guide needle was inserted from the sole of the foot towards the tibia, and a hollow drill was utilized along the guide needle opening after fluoroscopy confirmed the correct position of the guide needle (Fig. 1). Then, an initial surgical incision was made, and the induced membrane was carefully protected. The cement and the plate were removed, the end of the incision was carefully removed, fibrous tissue was used to freshen the bone tissue, and the cartilage was removed until the subchondral bone was reached. After pulse flushing with water and normal saline, the proximal tibia was reamed from the opening of the plantar fascia, a suitable length of intramedullary nail was chosen, the ankle and hindfoot force line was reconfirmed, and a screw was placed in the calcaneus, talus, and proximal tibia and locked in turn. Finally, the autograft bone was placed in the defects. For those with insufficient bone mass, allograft materials were mixed.

### Postoperative management and follow-up

X-ray examination was performed 1 day after the first stage, routine blood tests, liver and kidney function tests, and erythrocyte sedimentation rate (ESR) and C-reactive protein (CRP) measurements were performed at 1, 3, and 7 days. Antibiotics to which the isolates were sensitive were administered intravenously for 2 weeks. The negative pressure drainage tube was applied for 10–12 days, patency was observed, and the drainage volume was recorded. Antibiotics were used prophylactically for 24–48 h after the second stage. Gradual weight bearing was allowed 4 weeks after bone grafting, and full weight bearing was allowed when bone union.

## Results

There were 9 cases of distal tibia infection, 3 cases of both distal tibia and fibula infection, and 3 cases of talus infection. There were 5 cases on the right side and 10 cases on the left side. The initial causes of injury included 8 open fractures, 5 closed fractures, 1 snake bite, and 1 acupuncture injury. Patients received an average of 2.7 (0–6) operations before admission to our hospital. The average duration of bone infection was 5.6 (2–212) months.

There were 10 patients with positive bacterial isolation, including 5 *Staphylococcus aureus* isolates, 2 *Staphylococcus epidermidis* isolates, 1 *Pseudomonas aeruginosa* isolate, 1 *Escherichia coli* isolate, and 1 *Klebsiella pneumoniae* isolate. One patient had a soft tissue defect in which a skin flap was performed.

**Figure 2:**
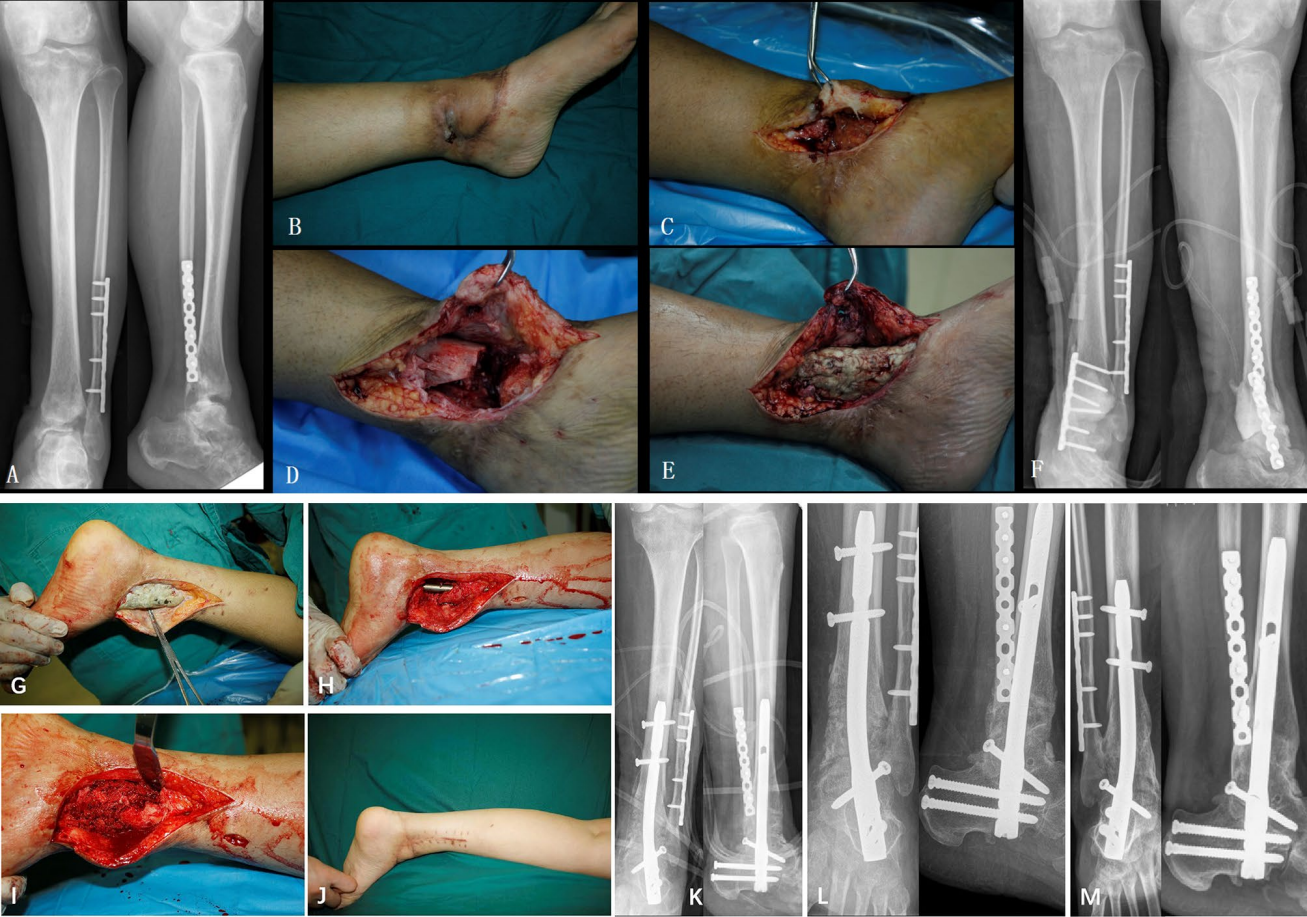
A 29-year-old female presented with repeated skin ulceration and pus of the left leg for 8 months. (**A**) Preoperative X-ray; (**B**) preoperative photograph; (**C**) pustules and necrotic tissue; (**D**) bone defects after debridement; (**E**) plate fixation and cement implantation; (**F**) postoperative X-ray; (**G**) pregrafting photograph showing no necrotic tissue; (**H**) retrograde intramedullary nail for ankle fusion; **I**: bone grafting; **J:** no ulceration or pus at the 3-month follow-up; (**K**) postoperative X-ray; (**L**) X-ray showing bone union occurring 6 months later; (**M**) bone union after 24 months.

Four patients experienced recurrence of infection after the first stage, which required debridement again, and one required local flap transfer to cover the wound. The length of the bone defect was 2.1–8.7 cm (average: 5.3 cm) after debridement. All patients were followed up for 18 to 42 months (average: 30 months). During the follow-up period, 2 patients experienced recurrence of infection. One patient was cured completely by debridement, and another patient underwent amputation under the knee due to uncontrolled infection. The remaining 13 patients (86.6%) achieved bone union without recurrence of infection, the average bone union time was 5.8 (4–10) months. No complications, such as internal fixation loosening, nonunion or calcaneal varus, occurred. The average ankle-hind foot function score (AOFAS) increased from 29.75 ± 4.37 to 81.06 ± 4.72 at the last follow-up (Table 1). Typical cases are shown in Fig. 2.

**Table 1:** AO-FAS score of the fifteen patients.

AOFAS Scores	Preoperative	Postoperative
Pain	12.52 ± 6.84	40.00 ± 0.00*
Activity limitations	2.55 ± 1.54	9.63 ± 1.05*
Maximum walking distance	0.93 ± 0.62	4.72 ± 0.34*
Walking surface	1.42 ± 1.61	4.12 ± 1.04*
Gait abnormality	3.21 ± 1.24	4.59 ± 1.14*
Sagittal mobility	0.82 ± 1.85	0.00 ± 0.00*
Hindfoot mobility	0.91 ± 1.43	0.00 ± 0.00*
Stability of ankle	2.66 ± 3.90	8.00 ± 0.00*
Alignment	4.73 ± 1.51	10.00 ± 0.00*
Overall score	29.75 ± 4.37	81.06 ± 4.72*

*P < 0.05.

## Discussion

Due to the lack of muscle coverage around the ankle, complications, such as soft tissue necrosis and nonunion, can easily occur, and the infection rate is as high as 10%^16^, which is a great challenge for orthopaedic surgeons. As a remedy to avoid amputation, ankle fusion is widely used in bone reconstruction for distal tibial infection with bone defects, ankle infection, and talus infection necrosis^1,11–13,17^. The purpose of surgical treatment is to effectively control infection, repair bone defects, restore painless and stable ankle joints that can support walking with full weight bearing^1^. The commonly used methods include Ilizarov technique^2,3^ and vascular bone grafting^6,7^, and each technique has its own advantages and disadvantages. At present, literature reports mostly using Ilizarov ring external fixator or hybrid (Hybrid) external fixator for joint fusion after infection^10–14^. The "distraction-stress principle" aims to repair bone defects, maintain the length of both lower limbs, and adjust the alignment of the hind feet. It plays an irreplaceable role in the repair of bone defects, but its large volume and pin site complications make it unbearable for many patients, thus limiting its application. The induced membrane technique is a relatively new technique, and the antibiotic cement implanted in the first stage is not only beneficial to the eradication of infection but can also promote the formation of an induced membrane around it, which promotes the repair of bone defects^4^, and its indications have expanded from initial traumatic bone defects to open fractures, nonunion, bone infection, congenital tibial pseudojoints, and tumours^4,8,9^. However, there are few reports on the treatment of ankle infections with a retrograde intramedullary nail combined with the induced membrane technique.

In this study, 15 patients with infected bone defects of the ankle were treated with the induced membrane technique. The cavity was filled with antibiotic bone cement after debridement, and antibiotic-loaded bone cement was used to wrap the locking plate^18,19^. Retrograde intramedullary nails combined with bone grafting in the membrane were used after the infection was controlled. We evaluated the clinical effects and functional recovery. In the last follow-up, 13 patients healed without infection, and the effect was good. We observed that the stability of the ankle was good, and most patients had significantly reduced pain and were satisfied with the recovery of ankle joint function. The AOFAS ankle-hind foot scores were significantly improved compared with the preoperative scores. Gessmann et al.^11^ reported 37 cases of ankle infections treated with the Ilizarov ring external fixator; the bone union rate was 94.6%, and the recurrence rate of infection was 5.4%, but the AOFAS functional score averaged 67.9 points. In our study, the bone union rate at the last follow-up was 86.6%, which was close to that in the previous report. The recurrence rate of infection was 13.3%, which was higher than that in previous literature.

Local stabilization plays an important role in infection control after thorough debridement, and common stabilization methods include Ilizarov or hybrid external fixators, plate screws, and intramedullary nails. The external fixator is placed over the infected area, and wires or half-pins are inserted outside of the infection focus to avoid biofilm infection. Furthermore, the bone transport technique can be used to repair bone defects, so it has always been regarded as the standard treatment, but external fixators have complications, such as pin-track infection, partial weight bearing for a long period, and the inability of many patients to tolerate the procedure. Plates require excessive stripping of soft tissues, especially when the ankle lacks muscle coverage, which easily causes soft tissue irritation, leading to delayed wound healing, dehiscence, and infection. In addition, the plate has a barrier effect on grafts, which may result in absorption of the graft. As a central fixation, a retrograde intramedullary nail has strong axial and anti-rotation stability and can provide immediate stability; it allows patients to walk on the ground early and saves some bone mass.

Our results showed low complication rates, which may be due to the following reasons: first, we preserve the fibula as much as possible to maintain the length of the limbs during debridement; the patient can walk with partial weight bearing before grafting, and this is beneficial to prevent osteoporosis and surrounding soft tissue contractures. In addition, the fibula is not stripped and used as the bone graft source, which greatly reduces trauma, preserves the soft tissue blood supply and saves operation time.

The shortcomings of the present study are the small sample size, short follow-up time, and lack of control groups. However, our experience in the treatment of ankle infections with retrograde intramedullary nails combined with the induced membrane technique can provide useful information. In the future, multicentre, prospective studies are needed to further clarify the effectiveness and scientific nature of this treatment.

## Conclusions

The induced membrane technique combined with a posterior intramedullary nail is an effective method to treat infected bone defects of the ankle. It can significantly improve hindfoot function, early weight bearing and quality of life, but attention is required to reduce the rate of infection recurrence.

## Supplementary Information

Supplementary Information.

## Data Availability

The datasets used and/or analysed during the current study are available from the corresponding author on reasonable request.
